# Comparative Transcriptome Analysis of Organ-Specific Adaptive Responses to Hypoxia Provides Insights to Human Diseases

**DOI:** 10.3390/genes13061096

**Published:** 2022-06-19

**Authors:** Kuo-Sheng Hung, Shiow-Yi Chen, Pang-Hung Hsu, Bo-An Lin, Chin-Hua Hu, Cing-Han Yang, Tun-Wen Pai, Wen-Shyong Tzou, Hsin-Yu Chung

**Affiliations:** 1 Center for Precision Medicine and Genomics, Tri-Service General Hospital, National Defense Medical Center, Taipei 114, Taiwan; 2 Department of Bioscience and Biotechnology, National Taiwan Ocean University, Keelung 202, Taiwan; sherry0930@mail.ntou.edu.tw (S.-Y.C.); phsu@ntou.edu.tw (P.-H.H.); boanlin@gmail.com (B.-A.L.); chhu@mail.ntou.edu.tw (C.-H.H.); paristzou@gmail.com (W.-S.T.); 3 Biotools Co., Ltd., New Taipei City 221, Taiwan; 4 Department of Computer Science and Information Engineering, National Taipei University of Technology, Taipei 114, Taiwan; cinghanyang@gmail.com (C.-H.Y.); twp@ntut.edu.tw (T.-W.P.); 5 Department of Computer Science and Engineering, National Taiwan Ocean University, Keelung 202, Taiwan; 6 Cancer Vaccine and Immune Cell Therapy Core Laboratory, Chang Gung Memorial Hospital, Linkou, Taoyuan 244, Taiwan

**Keywords:** circadian rhythm, cell proliferation, cholesterol synthesis, dopamine, GABA, glucose metabolism, hypoxia, glutamate

## Abstract

The common carp is a hypoxia-tolerant fish, and the understanding of its ability to live in low-oxygen environments has been applied to human health issues such as cancer and neuron degeneration. Here, we investigated differential gene expression changes during hypoxia in five common carp organs including the brain, the gill, the head kidney, the liver, and the intestine. Based on RNA sequencing, gene expression changes under hypoxic conditions were detected in over 1800 genes in common carp. The analysis of these genes further revealed that all five organs had high expression-specific properties. According to the results of the GO and KEGG, the pathways involved in the adaptation to hypoxia provided information on responses specific to each organ in low oxygen, such as glucose metabolism and energy usage, cholesterol synthesis, cell cycle, circadian rhythm, and dopamine activation. DisGeNET analysis showed that some human diseases such as cancer, diabetes, epilepsy, metabolism diseases, and social ability disorders were related to hypoxia-regulated genes. Our results suggested that common carp undergo various gene regulations in different organs under hypoxic conditions, and integrative bioinformatics may provide some potential targets for advancing disease research.

## 1. Introduction

Low oxygen affects life cell functions and human health when the body’s oxygen concentration changes to hypoxia (O_2_ lower than 1%). For example, hypoxia has long been noted as a hallmark due to its multiple roles in cancer development and neurodegeneration. Previous studies have indicated that hypoxia creates an imbalance between O_2_ consumption and availability in solid tumors [1]. The high metabolic change in cancer cells formed a hypoxic microenvironment that then caused changes in the expression and stability of some special genes such as hypoxia-inducible factors (*HIFs*), which affected cancer survival and progression by facilitating angiogenesis [2]. Studies have also indicated that in the hypoxic tumor microenvironment, cancer cells switched glucose metabolism to the pentose phosphate pathway and limited mitochondrial reactive oxygen species (ROS) accumulation and oxidative stress, resulting in cell survival [3]. Hypoxia also affects neuron and brain development and causes degenerative diseases. Yasui et al. found that human neural precursor cell derived from Rett syndrome patients could be promoted to create astrocytes, which harmed neuronal development under hypoxic conditions [4]. Our previous study also found that hypoxia in neural progenitor cells promoted neural differentiation mediated by the regulation of *BIRC5A* [5].

Hypoxia can also affect fish health and behavior; thus, hypoxia can be applied as a useful tool for evaluations in genetic and physiological studies. For this purpose, the common carp (*Cyprinus carpio*) is suitable due to its hypoxia-tolerance ability, which has been identified and discussed [6,7]. Moreover, some studies have demonstrated that the metabolism and gene changes in common carp under hypoxic conditions, such as abnormal levels of plasma glucose [8], and the upregulation of some hypoxia-induced marker genes, such as hypoxia inducible factor 1α (*HIF1A*), myoglobin 1 (*MO1*), and erythropoietin 2 (*EPO2*), could be detected [9].

In this study, we examined global gene expression changes by applying RNA sequencing (RNA-seq) to five common carp organs, i.e., the brain, the gill, the head kidney, the intestine, and the liver, under both normoxic and hypoxic conditions. According to the transcriptome analyses, our investigation revealed that each carp organ showed different gene expression regulation associated with specific biological and metabolic pathways such as energy production, cholesterol usage, cell proliferation, and glutamate signaling. These results provided a comprehensive view of the complex molecular events involved in the response of fish, in different organs, to environmental hypoxia stress and expanded our understanding of the response-to-hypoxia mechanism and its related diseases.

## 2. Materials and Methods

### 2.1. Hypoxia Treatment and Sampling

Common carp (*Cyprinus carpio*) were obtained from a local fish farm and reared in the laboratory for one month before the experimental treatment (total average length 35 cm, total average mass 650 g). Six fish were separated into hypoxia group (n = 3) and control group (n = 3). For hypoxia induction, three fish were transferred to each of two 35 l exposure tanks and left overnight in flow-through, aerated well-water. Hypoxia was achieved by covering the tank with Styrofoam and bubbling the water with nitrogen constantly. The content of dissolved oxygen decreased to 2.14 mg O_2_ L^−1^ over the first 2 h of bubbling and then was maintained at 2.14 mg O_2_ L^−1^ for the duration of the hypoxic exposure. The fish were removed from the tank after 72 h of hypoxia. The removed fish were immediately euthanized by neutralized MS-222 (100 mg L^−1^) and terminally sampled. The brain, fourth gill arch, liver, head kidney, and intestine were dissected, rinsed, and immediately frozen in liquid nitrogen and then stored at 80 °C until the subsequent analysis.

### 2.2. RNA Extraction

Total RNA was extracted from the organs with TRIzol (Invitrogen, Carlsbad, CA, USA), according to the following protocol: 1. Mixed 1 mL TRIzol, 0.5 mL isopropanol, and 100 mg organ; then they were homogenized using a homogenizer. 2. Incubated at 4 °C for 10 min and centrifuged at 12,000× *g* for 10 min. 3. Discarded supernatant and added 1 mL 75% ethanol. 5. After vortexing 10 s, centrifuged at 12,000× *g* for 5 min at 4 °C. 6. Discarded supernatant and air-dried samples for 10 min. 6. Added 50 μL RNAase-free water and suspended RNA pellet and incubated in heat block. 

### 2.3. Reverse Transcription

RNA was used to synthesize cDNAs with Superscript-II reverse transcriptase (Invitrogen, CA, USA) according to the following method: 1. Mixed 5 μg total RNA with 10 μg oligo (dT) primer, 0.5 mM dNTP, and RNAase-free water. 2. Incubated mixture at 65 °C for 5 min and chilled on ice. 3. Added first-strand buffer (to 1×), 0.01 M DTT and incubated at 42 °C for 2 min. 4. Added 200 units of Superscript-II reverse transcriptase and incubated at 42 °C for 50 min. 5. Stopped RT reaction by heating at 70 °C for 15 min. Before RNA sequencing, β-actin gene (*ACTB*), a widely used housekeeping gene, was detected by qPCR to confirm each sample’s *ACTB* expression was equal. 

### 2.4. Illumina RNA Sequencing

The cDNA library was constructed and then produced 150 bp length paired-end (PE) reads by NextSeq 500 Sequencing System (Illumina, San Diego, CA, USA). All cDNA library construction and sequencing steps were performed by a local sequencing company (Genomics, New Taipei City, Taiwan). Of the reads obtained from the sequencer, the low-quality reads (Q score < 20) were removed by Trimmomatic [10], and the sequence alignment was processed in the next step. All sequence information was uploaded to the NCBI BioProject database under the submission SRA ID PRJNA822546. Read alignment was performed for the Chinese common carp sequences with annotated gene information as a reference [11]. Read short sequences were aligned and mapped to Chinese common carp sequences by using the R package Rsubread [12]: 1. Created a hash table index of the target genome from reference. 2. Reads aligned with index reference genome. 3. Determined exon–exon junctions from alignment. Gene symbol was annotated by organism-level R package. PCA plot of presenting gene expression was created by ggplot2 package in R.

### 2.5. Gene Expression Analysis and Statistical Tests

Calculations of gene expression values were based on counting reads in sequence from RNA sequencing: read counts obtained from alignment analysis were applied to the gene expression values. The comparison of gene expression in hypoxia and normoxia groups was shown as fold changes, which means average hypoxia expression values over average normoxia expression values among triple repeat samples (n = 3 per organ). Quasi-likelihood F-test was used for statistical tests. All gene expression analysis operations were performed using edgeR [13]. After edgeR analysis, both *p*-values and false discovery rate (FDR) < 0.05 were employed for selecting genes with statistical significance.

### 2.6. Gene Functional Investigations

In the GO, KEGG, and human disease (DisGeNET) enrichment analyses, significant genes of each common carp organ with fold changes > 2 or <0.5 were selected and uploaded to DAVID [14] website (https://david.ncifcrf.gov/ (accessed on 12 August 2021)), and human genes were applied as the reference species. GO similarity network was operated by NaviGO [15] (https://kiharalab.org/web/navigo/views/goset.php (accessed on 4 September)) with GO terms obtained from DAVID, which had Benjamini-adjusted *p*-values < 0.05. Co-expression KEGG network analysis was constructed by Cytoscape software [16] with KEGG terms obtained from DAVID, which had Benjamini-adjusted *p*-values < 0.05.

## 3. Results

### 3.1. RNA Sequencing of Five Organs of Common Carp in Normoxia and Hypoxia

To determine organ-specific gene expression in common carp under hypoxic condition, we collected total RNA extracted from the brain, the gill, the head kidney, the intestine, and the liver from common carp treated under hypoxic conditions for 72 h or untreated (control) and detected the gene expression by using RNA-seq. Table 1 shows that the a total of 598 million 150 PE short sequence reads were generated by an Illumina NextSeq instrument, and on average, there were more than 20 million reads for each sample. The low-quality reads (lower than Q20) were removed, and the remaining reads were trimmed to an average length 143 bp. The trimmed sequences were aligned, and the sequences were further assembled by using the Chinese common carp genome as a reference template. After 88.9% of the reads were successfully assembled, an average of 57.2% of the sequences were annotated as genes for gene expression and function analyses.

### 3.2. Organ-Specific Transcriptome Analysis of Five Organs of Common Carp

**Figure 1:**
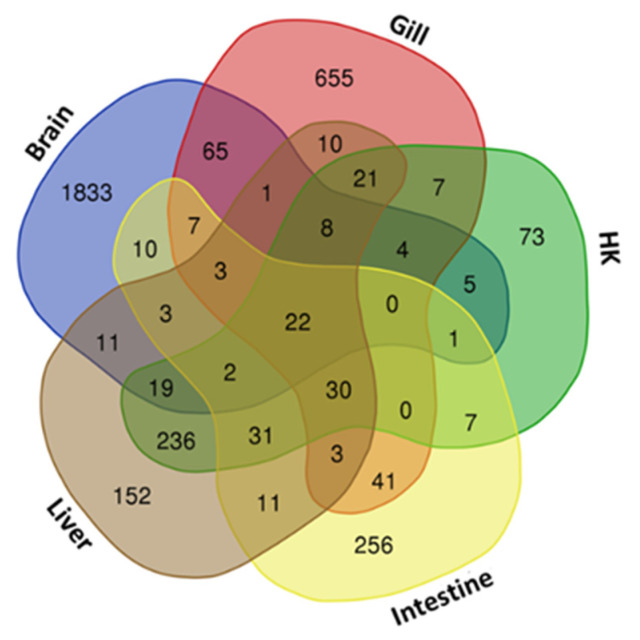
Venn diagram of gene sets in common carp treated under hypoxic conditions: Overlapping 22 genes in five organs are shown in the center, and each organ-specific gene is shown in the corners. HK: head kidney.

**Figure 2:**
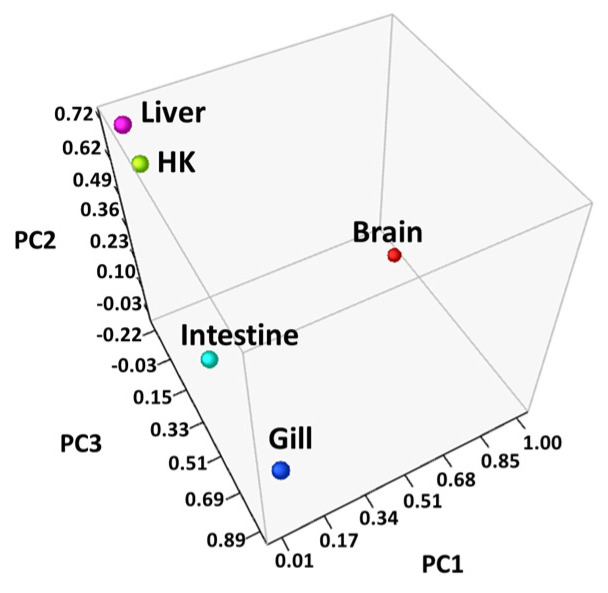
Principal component analysis (PCA) of the genes in all samples: Fold changes in each organ were converted to principal component values and visualized in three-dimensional coordinates, and the head kidney (HK) and liver were located closer to each other than the other organs.

Differentially expressed genes (DEGs) were identified through RNA-seq analysis (see Methods) for five organs (brain, gill, head kidney, liver, and intestine) of the common carp under normoxic and hypoxic conditions (Table S1). We found only 22 genes were expressed in all 5 organs (Figure 1). In addition, 1833 genes were expressed only in the brain, 655 genes were expressed only in the gill, and 152, 73, and 256 genes were expressed only in the head kidney, liver, and intestine, respectively. In the brain, we found the highest number of DEGs (1994 total, 1306 upregulated and 688 downregulated genes), followed by the gills (877 total, 477 upregulated and 400 downregulated genes), liver (563 total, 477 upregulated and 86 downregulated genes), head kidney (466 total, 375 upregulated and 91 downregulated genes), and intestine (427 total, 311 upregulated and 116 downregulated genes). The principal component analysis (PCA) of the fold changes in gene expressions in the five organs showed that the head kidney and liver had similar gene expression patterns, while the brain, gill, and intestine showed great differences (Figure 2), indicating that the organs in common carp had different responses and underlying mechanisms to adapt to the hypoxic environment. Further analysis of the 22 DEGs common in the five organs demonstrated that all were upregulated, and five of them (*DDIT4*, *EGLN1*, *EGLN3*, *ERO1A*, and *VEGFA*) were induced by hypoxia (Table 2). Gene *DDIT4*, *EGLN1,* and *EGLN3* in the head kidney and liver had very high fold changes in gene expression, as compared to those in the other organs, suggesting that *DDIT4*, *EGLN1*, and *EGLN3* played important roles in the physiological regulation in these two organs in a hypoxic environment.

### 3.3. Biological Function Analysis

The DEGs in each organ in a hypoxic environment were analyzed by DAVID based on the Gene Ontology (GO) database. A Benjamini-adjusted *p*-value less than 0.05 was applied as the threshold to select the matching GO biological process (GO BP) datasets (Table S2: upregulated genes, and Table S3: downregulated genes). From the comparison of the GO terms among the five organs, we discovered that, in the brain, liver and intestine, more GO terms were associated with upregulated genes. In contrast, in the gill, most GO terms were associated with downregulated genes. In the head kidney, GO terms were both evenly associated with up and downregulated genes.

**Figure 3:**
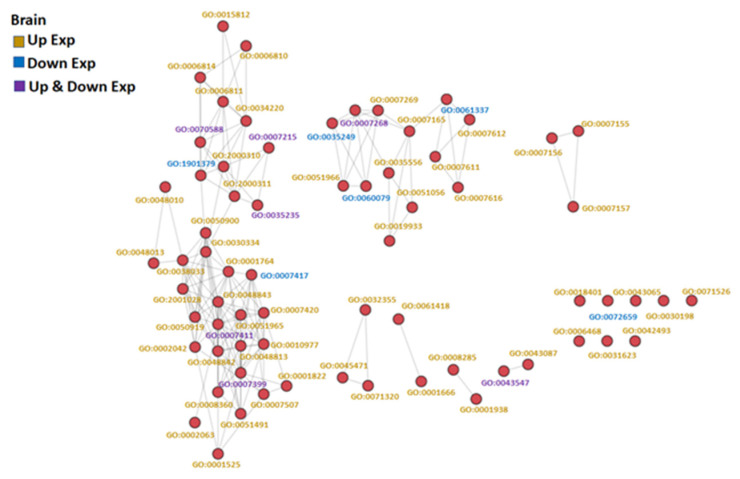
GO similarity network analysis of common carp brain under hypoxic conditions: The three similarity scores and three association scores were computed for each GO term pair, and a bubble network map was created.

**Figure 4:**
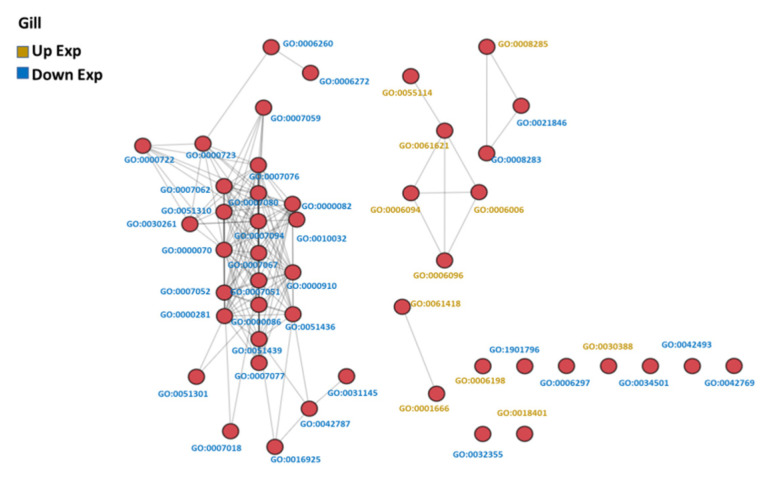
GO similarity network analysis of common carp gills in hypoxia: The three similarity scores and three association scores were computed for each GO term pair, and a bubble network map was created.

**Figure 5:**
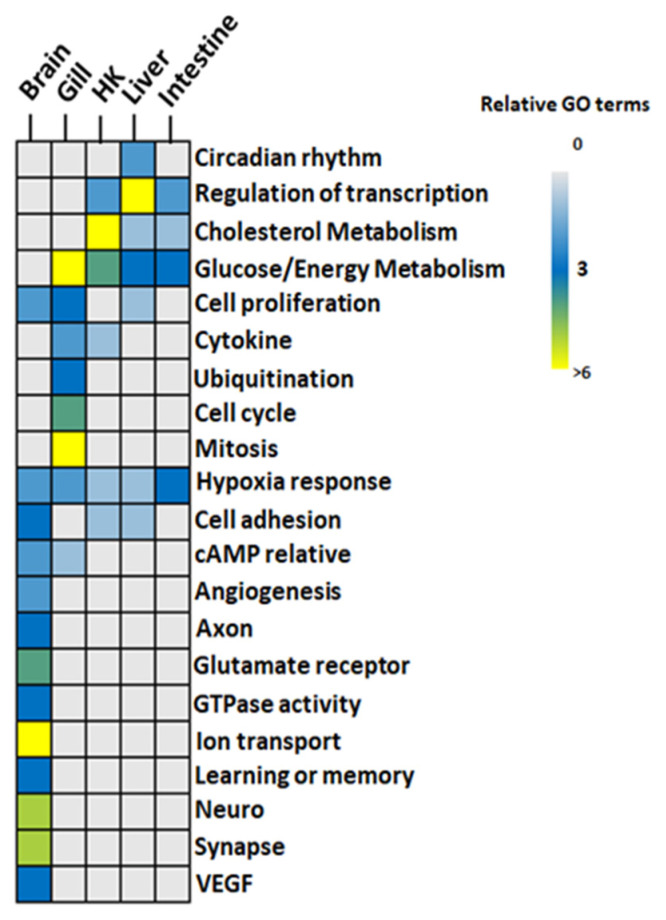
GO functions identified in hypoxia in five organs of common carp: GO terms from the GO similarity network were grouped. Color code: gray-to-blue (0–3 GO terms), blue-to-light green (3–6 GO terms), and yellow (>6 GO terms). HK: Head Kidney.

The GO-associated networks demonstrated that the brain and the gill had more complicated network structures (Figure 3 and Figure 4; GO term descriptions are provided in Tables S4 and S5), as compared to the other organs (Figure S1). The different responses of the organs to hypoxia were demonstrated by the significant differences in the major derived GO term categories of each organ (Figure 5), suggesting the different organs in common carp in a hypoxic environment coped with the hypoxic stress by regulating various biological processes in an organ-specific manner.

#### 3.3.1. Glucose Metabolism and Energy

**Figure 6:**
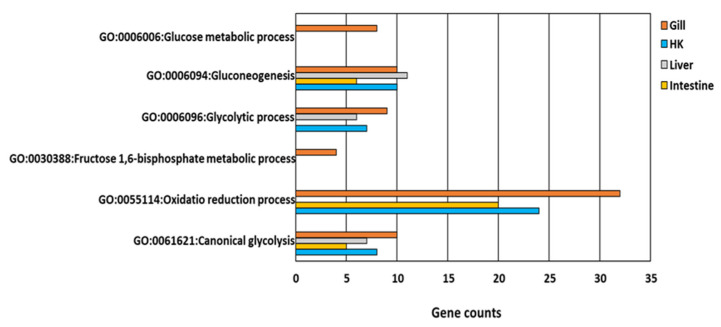
GO functions relative to glucose and energy metabolism in response to hypoxia in common carp organs: Four organs, including the gill, head kidney (HK), liver, and intestine, had the same GO terms in GO:0006094~gluconeogenesis and GO:0061621~canonical glycolysis.

All genes involved in glucose metabolism and energy-related GO functions (Figure 5) were upregulated in the gill, the head kidney, the liver, and the intestine of common carp under hypoxic conditions. Additionally, only two GO terms, GO:0006094~gluconeogenesis and GO:0061621~canonical glycolysis, were common in the four organs (Figure 6). By screening genes related to gluconeogenesis and canonical glycolysis, we found that *ALODA*, a gene belonging to the aldolase family, had high expression levels that were increased in the head kidney and the liver (11.44-fold and 16.64-fold in head kidney and liver, respectively) (Table 2). The high gene expression of *ALDOA* in the head kidney and the liver suggested that the carp species in an oxygen-deficient environment increased the control of energy in these two organs to maintain the energy required for normal activities.

#### 3.3.2. Cholesterol Biosynthesis

**Figure 7:**
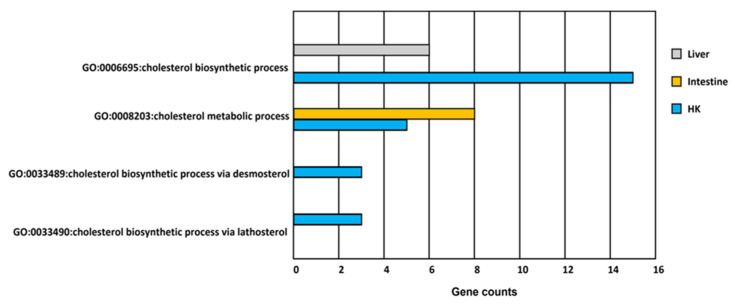
GO functions related to cholesterol metabolism in response to hypoxia in common carp organs. There are more than three genes involved in four cholesterol GO terms in the head kidney (HK). However, there is only one cholesterol GO term involved in the liver and the intestine.

The head kidney, liver, and intestine had cholesterol biosynthetic-related GO terms (Figure 5 and Figure 7). The expression of genes related to cholesterol biosynthesis in the head kidney were all decreased in hypoxia (Table S6). Furthermore, the expression levels of the genes *APOA4*, *EBP*, *FDFT1*, *FDPS*, *HSD17B7*, *MSMO1*, and *TM7SF2* were suppressed in hypoxia to less than 10% of those in normoxia, showing that cholesterol synthesis had been strongly inhibited in the head kidney in a hypoxic environment. 

#### 3.3.3. Transcription and Cell Cycle

In addition, many upregulated genes were related to the regulation of transcription, such as the activation of the RNA polymerase II promoter and circadian regulation (also called circadian rhythm) in the liver. In the gill, a large number of genes were related to the inhibition of cell proliferation, cell cycle, cell division, and mitosis, indicating that the cell proliferation was repressed in the gills under hypoxic conditions. Furthermore, the expression of the ubiquitination-related genes in the gill was also inhibited.

#### 3.3.4. Neuron Activity

Finally, the brain had the most unique physiological effects (Figure 5) related to the axons, learning/memory, cell adhesion, glutamate receptors, ion transport, and synaptic reactions. Other functional items related to neural signal transmission were reflected in the involvement of GTPase. In addition to the signal transmission of neurons, the genes characterized by angiogenesis functions and vascular endothelial growth factor (VEGF) were also a part of the carp brain response to hypoxia. Overall, the GO analysis results showed that the carp brain had the most complex regulation patterns. Further analysis showed that among these GO terms, 102 genes were involved in two GO terms, while only 54 genes were involved in more than three GO terms (Table S7). Additionally, 14 of these 54 genes (i.e., *ADCY1*, *CTGF*, *EFNA5*, *GATA3*, *GRIA1*, *GRIN1*, *GRIN2B*, *GRM4*, *GRM5*, *NECTIN1*, *NRP2*, *PCDH18*, *PRKD1*, and *SYT1*) showed an increase of more than 8-fold (Table 3). This result fully demonstrated that gene regulation may have a diverse mechanism in the carp brain under hypoxic conditions.

### 3.4. Pathway Analysis

#### 3.4.1. HIF-Related Pathways

**Figure 8:**
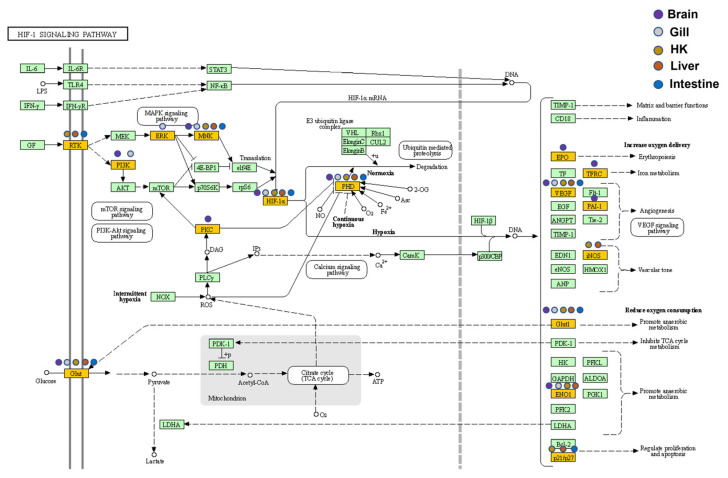
Expression patterns of the *HIF-1* signaling pathway in five common carp organs: All target genes are colored in gold, and organ-specificity is shown at different points. Purple: brain, gray: gill, orange: head kidney (HK), red: liver, and blue: intestine.

An enrichment pathway analysis (Table S8: upregulated genes and Table S9: downregulated genes) of genes was conducted using DAVID through the KEGG pathway database. After the characterization of the KEGG terms under a Benjamini-adjusted *p*-value less than 0.05 as described above, the genes involved in the *HIF-1* signaling pathway were identified in all five organs (Figure 8). In addition to the DEGs common in five organs (e.g., *HIF1A*) in this pathway, DEGs such as *EPO*, *PKCB*, and *TFRC* were only found in the carp brain, and their downstream genes were associated with GO terms such as angiogenesis and VEGF. The downstream genes of the *HIF-1* signaling pathway in the head kidney and the liver related to the functions of increasing oxygen transport and reducing oxygen consumption had similar fold changes in gene expression during hypoxia (Table 4), and notably, *NOS2* and *SLC2A1* showed very high increases in gene expression in hypoxia (more than 50-fold) in the head kidney and the liver.

#### 3.4.2. Glycolysis and Energy

In the gill, the head kidney, the liver, and the intestine, the upregulated genes were found to be involved in the pathways of glycolysis/gluconeogenesis (Figure S2). These genes represented 12 different functional families of glucose metabolism and were widely present in the fish gill, head kidney, and liver. Further analysis of other related pathways related to energy utilization revealed that these four organs employed different pathways to participate in energy usage (Table 5). For example, the liver and the intestine used the *AMPK* signaling pathway and *FoxO* signaling pathway during hypoxia while the head kidney, the liver, and the intestine used the *PI3K-Akt* signaling pathway during hypoxia. These findings suggested that the common carp could enhance energy metabolism by facilitating different pathways in a hypoxic environment.

#### 3.4.3. Steroid Biosynthesis

**Figure 9:**
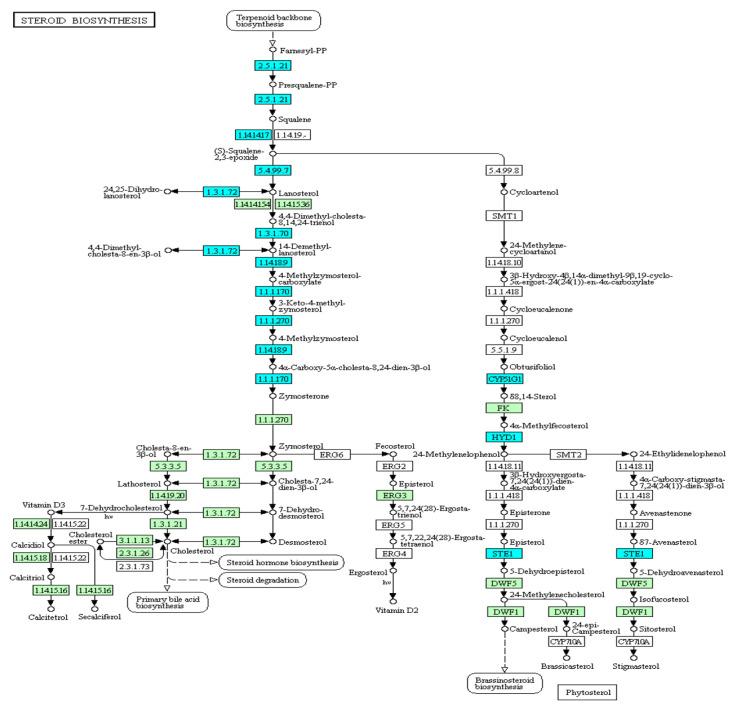
Expression patterns of the steroid biosynthesis pathway in common carp head kidney: All target genes are colored in blue.

The steroid biosynthesis pathway was suppressed in the head kidney (Figure 9), which was consistent with the strong inhibition of cholesterol metabolism found in the GO analysis. In total, 11 genes expressed in this pathway were inhibited, and 8 genes (*DHCR24*, *FDFT1*, *HSD17B7*, *LSS*, *MSMO1*, *NSDHL*, *SQLE*, and *TM7SF2*) were related to the production of the important upstream intermediate product, zymosterol. Therefore, this inhibitory mechanism of the head kidney was speculated to reduce damage to common carp caused by steroid production in a hypoxic environment.

#### 3.4.4. Cell Cycle

In the gill, the gene expression of the cell cycle (Figure S3) and DNA replication (Figure S4) were inhibited (16 genes were associated with the cell cycle, and 10 genes were associated with DNA replication). In the cell cycle, the genes with suppressed expression in the gill traversed the G1, S, G2, and M phases through downregulated *CYC*, *CDK*, and *MCM* gene expression. Moreover, *Cdc20*-related ubiquitin-mediated proteolysis was observed to be regulated in the cell cycle, which is consistent with the term ubiquitin-related effects found in the GO analysis. The DNA replication pathway in the gill reflected the suppression of eukaryote replication complex-related proteins such as DNA polymerase, *MCM* (DNA helicase), *RPA* (DNA replication protein A), and clamp. DNA polymerase ε1, ε2, ε3, and ε4 complexes responsible for the synthesis of the leading strand in the role of DNA polymerase in synthesizing new strands of DNA were also found to be inhibited, while in the *MCM* complex, half of the subunits (*MCM* 2, *MCM* 5 and *MCM* 6) were inhibited in expression. Two genes (*RFA1* and *RFA2/4*) in the three *RPA* subunits were also inhibited in expression. These phenomena of DNA replication inhibition combined with the inhibition of the cell cycle could indicate a unique mechanism in carp gills to affect their cell division and growth under hypoxic conditions in order to improve the efficiency of respiration and oxygen uptake.

#### 3.4.5. Pathway Interaction Network

**Figure 10:**
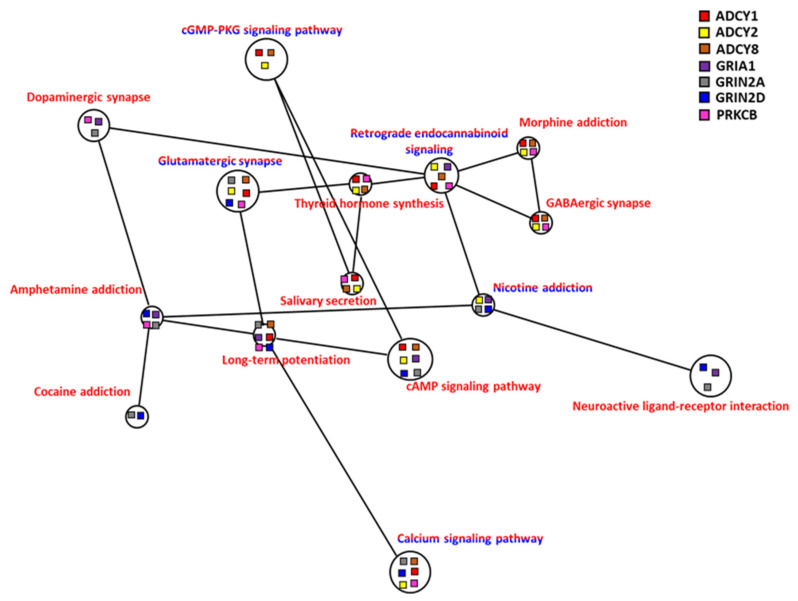
Correlation of co-expression KEGG network of brain of common carp in hypoxia: Co-expression genes including upregulated genes only (red lettering) and both up/downregulated genes combined (red/blue lettering) were analyzed. The node of each connected gene (*ADCY1*, *ADCY2*, *ADCY8*, *GRIA1*, *GRIN2A*, *GRIN2D*, and *PRKCB*) in the KEGG network is shown as a square.

In a hypoxic environment in the carp brain, there were very diverse pathways, and as many as 32 different KEGG pathways met the Benjamini selection conditions. Using NaviGO to further explore whether these pathways were related to each other, there were 7 genes present in 14 pathways that had formed a multiple pathway interaction network (Figure 10). The seven genes that constituted this network, *ADCY1*, *ADCY2*, *ADCY8*, *GRIA1*, *GRIN2A*, *GRIN2D*, and *PRKCB*, had specific expression patterns in the carp brain and played an important role in the operation of the brain and the mechanism of nerve conduction. Our scrutiny of the interaction network showed that common carp could activate the cGMP-related pathway (cAMP signaling pathway and cGMP-PKG signaling pathway), which was related to calcium ion signaling in a hypoxic environment. After the common carp were treated with hypoxia, the genes related to calcium ion signaling showed both increased and decreased expression. The downstream signaling pathway was also related to the long-term potentiation pathway, which is related to learning and memory. We speculated that carp could employ these mechanisms to maintain their cognitive learning abilities when in a hypoxic environment. In addition, this network was also involved in the synthesis of thyroxine and the pathway of salivary secretion. The regulation of thyroid hormone and salivary secretion may allow common carp to achieve the best oxygen-use efficiency by increasing the intake of oxygen or reducing the consumption of oxygen

#### 3.4.6. Dopamine and Endorphin

A regulatory mechanism composed of dopamine-related chemicals (amphetamine, cocaine, dopamine, morphine, nicotine) in the central nervous system was identified on the network map (Figure 10), and these pathways were associated with the GABAergic synapse, glutamatergic synapse, and retrograde endocannabinoid signaling. These endorphins play crucial roles in pathways such as long-term potentiation, neuroactive ligand-receptor interaction, and thyroid hormone synthesis, and thus have a significant effect on brain function, memory, and learning ability in carp during hypoxia.

### 3.5. Human Disease Gene Annotation

We collected DEGs in the analysis of GO and KEGG pathways, as shown above, and applied to the DisGeNET database for an analysis of the genes associated with human diseases. DisGeNET results are listed in Table S10 (genes selected from the GO analysis) and Table S11 (genes selected from the KEGG analysis). We identified some DEGs (from the brain of common carp) significantly associated with human diseases (Benjamini-adjusted *p*-value < 0.05), including epilepsy, depressive syndrome, and bipolar disorder (Table 6 and Table 7). The DEGs from the other organs showed genes related to cancer or metabolism, such as liver carcinoma, glycogen storage disease, and diabetes. Interestingly, the genes collected from the head kidney were associated with Q fever (*DHCR24*, *HMGCR*, *LDLR*, and *LSS*), a disease caused by the infection of *Coxiella burnetii* (Table 6). In addition, in the common carp liver, the KEGG pathway analysis showed that Alzheimer’s disease was related to genes *INSR*, *GAPDHS*, *BAX*, *DHCR24*, *PPARG*, *ENO1*, and *VEGFA* (Table 7). These results indicated that an understanding of hypoxic adaptation in common carp could provide useful information for human diseases.

## 4. Discussion

### 4.1. Hypoxia, HIF, and Energy Regulation in Common Carp

Previous studies have shown that common carp have a high tolerance to hypoxia [17], and its survivable ambient dissolved oxygen (DO) can reach as low as 0.5 mg/L [18]. To investigate the gene expression of common carp in response to a hypoxic environment and the underlying mechanisms, we applied high-throughput sequencing technology for the analysis of gene expression changes and the corresponding bio-functions. Earlier studies showed that myoglobin in the blood of fish increased under hypoxic conditions to enhance the use of oxygen in carp [19]. In this study, we also observed that in the carp gill, myoglobin gene expression was upregulated by 5.1-fold. The central pathway response induced by hypoxia involves the regulation of *HIF*-related genes. In this study, five DEGs (*DDIT4*, *EGLN1*, *EGLN3*, *ERO1A*, and *VEGFA*) common to five organs after the hypoxia treatment were discovered, and these genes are all related to the *HIF* pathway. *DDIT4* (also called *REDD1*) has been reported to be upregulated and promote apoptosis by inhibiting the mTOR pathway when cells were under stress such as DNA damage and hypoxia [20]. *DDIT4* has also been detected in participation with autophagy in dopaminergic neurons [21] as well as in development effects [22]. The *EGLN* family (also called *PHD*, prolyl hydroxylase)has been characterized as an oxygen-sensing gene and is usually suppressed in hypoxia; however, our results showed its high expression. It was clear that *EGLN* had inhibited *HIF* expression [23], and previous studies of common carp have shown that hypoxia would not inhibit *EGLN* activity. Therefore, the effects of upregulation in *DDIT4* were probably through the upregulation of the *EGLN-HIF* system, especially in the head kidney and the liver.

Recently, studies have shown that many fish have *HIF* regulatory systems [24,25]. To further examine the molecular mechanism underlying the pathway in response to hypoxia, the promoter sequences of 22 DEGs were investigated to determine the enriched binding sites of transcription factors. *HIF1A* bindings that appeared in 14 genes (*p* value < 1 × 10^−4^) in the 2000 base pairs of the upstream sequences of 22 DEGs were common to five organs (data not shown). In addition, some studies have shown that the regulation of *HIF* in fish was related to genes regulating glycolysis and glucose transport [26,27]. Glucose consumption caused by an anaerobic environment has been reported in many tumor biological studies and widely discussed in the Warburg effect [28]. In cancer cells, glycolysis increases with decreased oxygen availability, offering a benefit for growth [29]. Recent studies have demonstrated that the key enzyme *ALDOA* (aldolase A) in glycolysis is a cancer-related gene according to the Human Protein Atlas information [30]. *HIF1A* was also reported to upregulate *ALDOA* expressions under hypoxic conditions [31]. *HIF* has also been shown to enhance the glucose flux in glycolysis in cancer cells to enhance energy usage [32]. Zhu reported that in addition to *HIF*-related pathways, the involvement of *AMPK*, *PI3K-Akt*, and insulin-related signaling pathways in fish played an important regulatory role in hypoxia [33]. In the same vein in this study, significant changes were found in the expression levels of *AMPK* and *PI3K-Akt*, which are connected to obesity and diabetes [34,35], as well as the insulin signaling pathway-related genes in the liver and the intestine, indicating that common carp have a complex set of gene regulation networks in response to the influence of a hypoxic environment.

### 4.2. Hypoxia Affects Cholesterol-Related Biosynthesis in the Head kidney of Common Carp

Van Raaij et al. suggested that, in addition to lactate, the stress-related substance cortisol (synthesized from cholesterol) was found in carp blood under hypoxic conditions [36,37]. Through the results published by Van Raaij et al. in 1996, it was found that the carp had excessive glucose accumulation (hyperglycemia) in the blood in an environment of long-term hypoxia, which was believed to result from the glycogenolysis in the liver caused by stimulation via catecholamines or cortisol [38,39]. In addition, in 1994 and 2001, Van Raaij et al. suggested that lactate and catecholamines accumulated in carp blood [40,41]. In 2000, the study by Zhou et al. also suggested that the livers in common carp had a large amount of lactate when measured under hypoxic conditions [42]. In 2014, Moyson et al. suggested that the livers of common carp could use Cori cycle under hypoxic conditions to convert glycogen to lactate for energy production, and lactate could be synthesized back into glycogen after sufficient energy was available [43].

Cholesterol-related biosynthesis studies have also indicated that in a hypoxic environment, the activity of HMG-CoA reductase (*HMGCR*), an enzyme that synthesizes cholesterol in common carp, decreased, and the level of high-density cholesterol (HDL) in the blood decreased [44]. In this study, it was found that the gene expression of HMGC in the head kidney and the intestine of the carp was significantly decreased (88% during hypoxia), and the other gene-encoding enzymes for the biosynthetic pathway of cholesterol were also downregulated. These studies and our findings suggested that carp adapt to hypoxia by regulating the glycolysis/gluconeogenesis pathway (e.g., via the upregulation of *ALDOA*), reducing cholesterol biosynthesis to increase the production of catecholamines or cortisol and further ensuring ATP production during hypoxia.

In recent years, the roles of cholesterol in the immune system have been discussed. Cardoso et al. indicated that the level of cholesterol could respond to immune responses (including innate and adaptive immune cell systems) [45]. Intriguingly, a gene of *C. burnetii* encoding a sterol-modifying enzyme was determined to be critical for the survival in phagolysosome-like vacuoles and essential for pathogenesis [46]. Therefore, our study demonstrated how common carp adapted to hypoxia by regulating cholesterol synthesis and thereby modulating the immune system and pathogenic resistance.

### 4.3. Common Carp Gill, Liver, and Intestine Exhibit Different Functional Changes under Hypoxic Conditions

According to the research results reported by Dhillon, the shape of the gills changed during hypoxia by reducing the interlamellar cell mass (ILCM) to increase the surface area of the gills, thereby increasing oxygen uptake [47]. Nilsson reported that ILCM changes were achieved based on increasing apoptosis and inhibiting mitosis [48]. In this study, many mitosis genes were downregulated in the gills during hypoxia. Moreover, genes involved in cell proliferation, the cell cycle, and DNA replication were suppressed. The cell cycle arrest of human and mouse cell lines in hypoxia has been reported [49,50]. Furthermore, the growth of normal cells was inhibited in hypoxia through cell cycle arrest, but it rarely affected tumor cells [51]. According to research reports, hypoxia inhibited *HIF*-related gene ubiquitination, such that *HIF* accumulated in large quantities without being decomposed in the nucleus, which continuously stimulated the expression of downstream genes [52,53]. In this study, we found that the expressions of many ubiquitination-related genes were inhibited in the gill such that common carp increased the efficiency of oxygen utilization and the use of energy in an oxygen-deficient environment.

The specific growth rate (SGR) of carp under hypoxic conditions was reported to be only 70% of that under a normal growth environment [54], revealing that the growth of carp could be related to the *FoxO* signaling pathway [55]. This finding was consistent with the upregulation of genes related to the *FoxO* signaling pathway in the liver and intestine of carp during hypoxia in our study. It has been also reported that diabetes and obesity could be associated with the *FoxO* signaling pathway due to its role in glucose balance regulation [56]. Glucose consumption under hypoxic conditions in common carp may provide a novel view from which to study diabetes.

We found that some DEGs in the liver of common carp were responsible for the circadian rhythm. A study of obstructive sleep apnea (OSA) in humans reported that the *HIF1A* protein level increase could damage the circadian clock [57]. It was suggested that sleep deprivation and circadian rhythm disruption may increase the development of Alzheimer’s disease [58]. Bassendine et. al. showed that amyloid-β (Aβ), the main biomarker of Alzheimer’s disease, was originally generated in the liver [59]. Aβ also functions through the liver and is involved in the balance of circulating Aβ in the blood. It was also suggested that circadian rhythms, type 2 diabetes, and Alzheimer’s disease were linked in humans [60]. Our research results were in the same vein and suggested that carp could be a good model in corresponding research regarding diagnosis and therapeutic treatment.

### 4.4. Common Carp Brain Showed Complex Gene Regulation under Hypoxic Conditions

The carp brain was shown to undergo anaerobic metabolism under hypoxic conditions to maintain the ATP content required for physiological needs [61]. From the analysis of the *HIF-1* signaling pathway, we found that the carp brain exhibited an increased expression in genes related to anaerobic metabolism. Our study also suggested that the gene expression related to cGMP-PKG, calcium signaling, and cAMP-related activity was regulated in the carp brain. Activating cGMP regulated the K_ATP_ channels under hypoxic conditions to reduce the amount of Ca^2+^ influx into cells and thereby store ATP [62]. However, from the investigation of the cortisol synthesis pathway, it was found that the cAMP and calcium signaling pathways were upstream of the cortisol synthesis pathway; thus, the regulation of these two pathways could help common carp produce cortisol in order to help control energy consumption under hypoxic conditions. It was also reported that under hypoxic conditions, the carp brain activated nitric oxide synthase (*NOS*) by Ca^2+^ to reduce NO damage to the brain [63]. The regulation of cAMP was confirmed to affect learning and memory in a study of brain hypoxia in zebrafish. Moreover, the regulation of cAMP in carp prevented hypoxia from damaging its cognitive learning and memory functions [64]. Delhaye and Bardoni reported that the phosphodiesterases (PDEs) family played a major role in cAMP and cGMP production and was involved in neurodevelopmental disorders [65], such as depressive syndrome and autistic disorder, which was consistent with our results that *PDE4B* had 2.7-fold upregulation, on average, and also was involved in the hsa04024:cAMP signaling pathway (Tables S1 and S8).

The production of GABA and glutamate was demonstrated to improve the survival rate of the brain by reducing metabolic consumption [66]. In 2013, Lardon et al. used 1H-NMR to measure substances produced by carp brains treated with hypoxia, and after 24 h, the glutamate level of common carp in hypoxia was lower than in normoxia [67]. GABA was demonstrated to increase *HIF1A* and thyroid levels to further regulate metabolism in *Cirrhinus mrigala* under hypoxic conditions [68]. The interaction between cortisol and the thyroid was also shown to regulate ion transport and achieve the regulation of Na^+^/K^+^-ATPase [69]. Interestingly, in addition to the complex gene regulation patterns described above, the transcriptome analysis in our study inferred the regulation of amphetamine, cocaine, dopamine, morphine, nicotine, and retrograde endocannabinoid in the carp brain as the adaptation mechanism in a hypoxic environment. These pathways may also be linked to the regulation of GABAergic synapse and glutamatergic synapse. It has been reported that dopamine helped regulate Na^+^/K^+^ -ATPase [70]. A novel therapy of epilepsy focusing on targeting GABA and dopamine regulation has been discussed [71,72] and, in a study of Parkinson’s disease, dopamine was demonstrated to increase anti-oxidative damage in the blood and delay neurodegeneration [73,74]. Therefore, we hypothesized that when common carp were placed under hypoxic stress, the brain would first activate neuron-related genes, as described above, through the *HIF-1* regulation system and activate multiple neuron-related pathways via dopamine-related pathway regulation to avoid damage from hypoxic stress.

### 4.5. Limitation of the Present Study

Our research was based on a hypoxia-induced fish gene study combined with bioinformatics analysis, not a mammalian cell culture study or clinical patient data. Therefore, some genes with high transcription could not be annotated with functions that may be important in hypoxia regulation. These genes will be the focus of our next study to clarify their biological roles in pathways and their relationship to human health through experiments in model animals such as zebrafish (*Danio rerio*).

## 5. Conclusions

We employed high throughput RNA sequencing to examine gene expression changes and decipher organ-specific differences in transcriptomes under hypoxic conditions in common carp. The results showed that five common carp organs had various DEGs and functional patterns. The GO and KEGG pathways involved in the adaptation to hypoxia not only provided information on responses specific to each organ in low oxygen, such as glucose metabolism and energy usage, but also for cholesterol synthesis, cell cycle, circadian rhythm, and dopamine activation. Further findings in this study suggested many clues to the cellular mechanisms of human diseases (e.g., cancer, infection, neuron degeneration, social anxiety disorder, and metabolic syndrome). Using specific inhibitors against these hypoxia-induced proteins (e.g., *NOS2* inhibitor) or receptors (e.g., GABA) may provide new developments in future disease therapy.

## Figures and Tables

**Table 1:** Summary of RNA sequencing and alignment to Chinese common carp ^1^.

**Hypoxia**	**Brain**	**Gill**	**HK ^2^**	**Intestine**	**Liver**
Total paired reads	21,658,806	20,529,026	20,496,185	21,636,720	20,985,179
Mapped reads	19,444,227	18,177,160	18,223,421	19,106,839	18,627,812
Mapped percentage (%)	89.8	88.5	88.9	88.3	88.8
Successfully assigned alignments to gene annotation	10,465,513	10,873,662	13,137,988	12,236,594	13,490,836
**Normoxia**	**Brain**	**Gill**	**HK**	**Intestine**	**Liver**
Total paired reads	17,702,578	19,624,198	19,789,181	17,966,931	18,947,961
Mapped reads	17,671,462	17,354,099	17,611,558	15,885,049	16,880,884
Mapped percentage (%)	89.8	88.5	89.0	88.4	89.1
Successfully assigned alignments to gene annotation	9,257,355	10,473,164	12,716,294	10,175,030	12,270,737

^1^ These statistics were calculated by the average value of three replicates from each sample. ^2^ Head kidney.

**Table 2:** A total of 22 differentially expressed genes common in five organs of common carp under hypoxic conditions.

Sequence ID ^1^	Gene Symbol	Expressed Fold Change				
		Brain	Gill	HK ^2^	Liver	Intestine
LOC109048049	*EGLN3*	15.02	14.75	745.72	369.05	34.80
LOC109048372	*EGLN1*	4.70	36.86	377.67	123.97	17.58
LOC109053018	*IVNS1ABP*	3.80	3.24	6.78	8.36	4.73
LOC109056673	*SLC2A1*	3.36	4.00	75.36	97.45	6.66
LOC109057161	*HSPA1L*	4.49	5.42	24.77	9.48	8.31
LOC109065938	*VEGFA*	3.94	3.24	5.90	6.53	4.62
LOC109068950	-	12.29	5.71	46.28	27.97	17.89
LOC109069551	*GYS1*	3.01	8.87	10.62	8.52	6.00
LOC109070211	*MCL1*	8.58	2.56	12.45	15.01	4.83
LOC109071096	*MKNK2*	2.86	6.76	28.50	41.44	8.49
LOC109078178	*DDIT4*	7.20	11.28	151.68	141.12	18.09
LOC109079204	*VEGFA*	4.63	3.21	7.79	7.85	5.89
LOC109083134	*GABARAPL1*	2.68	6.36	8.50	10.06	8.38
LOC109084538	*HSPA1L*	4.24	4.99	36.79	12.55	11.29
LOC109085797	*CPOX*	3.66	6.10	6.75	5.95	4.71
LOC109088789	-	4.37	7.68	22.38	24.90	14.15
LOC109097019	*ALDOA*	2.64	2.79	11.44	16.64	4.37
LOC109104786	*ZNF395*	3.12	20.03	124.39	54.89	41.06
LOC109105991	*MCL1*	4.22	3.61	11.58	11.73	5.70
LOC109106889	*ERO1A*	2.64	6.55	33.63	28.75	9.08
LOC109108375	*PMM1*	4.78	3.67	47.20	13.16	12.96
LOC109113598	*GYS1*	2.80	11.61	8.36	7.88	8.22

^1^ Assembled with Chinese common carp. ^2^ Head Kidney.

**Table 3:** DEGs with >8 fold change in the common crop brain after hypoxia treatment.

Gene	Fold Change	Relative GO Terms
*ADCY1*	8.17	GO:0007616~learning or memory GO:0019933~cAMP-mediated signaling GO:0035556~intracellular signal transduction
*CTGF*	15.54	GO:0007155~cell adhesion GO:0035556~intracellular signal transduction GO:0001525~angiogenesis
*EFNA5*	8.19	GO:0007399~nervous system development GO:0007411~axon guidance GO:0051965~positive regulation of synapse assembly GO:0048013~ephrin receptor signaling pathway
*GATA3*	8.11	GO:0001764~neuron migration GO:0007411~axon guidance GO:0007165~signal transduction
*GRIA1*	21.26	GO:0007165~signal transduction GO:0007268~chemical synaptic transmission GO:0007616~long-term memory
*GRIN1*	10.78	GO:0007268~chemical synaptic transmission GO:0007616~long-term memory GO:0035249~synaptic transmission, glutamatergic GO:0048013~ephrin receptor signaling pathway GO:0060079~excitatory postsynaptic potential
*GRIN2B*	10.21	GO:0007268~chemical synaptic transmission GO:0007611~learning or memory GO:0048013~ephrin receptor signaling pathway
*GRM4*	8.03	GO:0051966~regulation of synaptic transmission, glutamatergic GO:0007268~chemical synaptic transmission GO:0007269~neurotransmitter secretion
*GRM5*	11.60	GO:0007268~chemical synaptic transmission GO:0007612~learning GO:0051966~regulation of synaptic transmission, glutamatergic
*NECTIN1*	9.50	GO:0007411~axon guidance GO:0007155~cell adhesion GO:0007156~homophilic cell adhesion via plasma membrane adhesion molecules GO:0007157~heterophilic cell-cell adhesion via plasma membrane cell adhesion molecules GO:0007165~signal transduction
*NRP2*	13.00	GO:0001525~angiogenesis GO:0007155~cell adhesion GO:0007411~axon guidance GO:0048010~vascular endothelial growth factor receptor signaling pathway
*PCDH18*	15.61	GO:0007155~cell adhesion GO:0007156~homophilic cell adhesion via plasma membrane adhesion molecules GO:0007399~nervous system development GO:0007420~brain development
*PRKD1*	45.28	GO:0001525~angiogenesis GO:0007165~signal transduction GO:0035556~intracellular signal transduction GO:0038033~positive regulation of endothelial cell chemotaxis by VEGF-activated vascular endothelial growth factor receptor signaling pathway GO:0048010~vascular endothelial growth factor receptor signaling pathway GO:2001028~positive regulation of endothelial cell chemotaxis
*SYT1*	222.49	GO:0007268~chemical synaptic transmission GO:0007269~neurotransmitter secretion GO:0007420~brain development GO:0051966~regulation of synaptic transmission, glutamatergic

**Table 4:** Fold changes of oxygen-related genes expressed in the *HIF-1* signaling pathway.

**Related to Increased Oxygen Delivery**					
**Gene**	**Brain**	**Gill**	**HK ^1^**	**Liver**	**Intestine**
*EPO*	6.48	-	-	-	-
*NOS2*	-	-	54.27	97.70	-
*TFRC*	4.61	-	-	-	-
*SERPINE1*	7.12	-	-	-	-
*VEGFA*	4.63	3.21	7.79	7.85	5.89
**Relayed to Reduced Oxygen Consumption**					
**Gene**	**Brain**	**Gill**	**HK**	**Liver**	**Intestine**
*CDKN1B*	-	-	3.06	3.40	5.25
*ENO1*	4.05	4.92	11.10	9.87	-
*SLC2A1*	3.36	4.00	75.36	97.45	6.66

^1^ Head Kidney.

**Table 5:** Energy-related metabolism pathways enriched in common carp.

KEGG Pathway	Gill	HK ^1^	Liver	Intestine
hsa00010:Glycolysis/Gluconeogenesis	V	V	V	V
hsa00030:Pentose phosphate pathway	V	-	-	V
hsa00051:Fructose and mannose metabolism	V	-	-	V
hsa01200:Carbon metabolism	V	V	-	-
hsa04068:FoxO signaling pathway	-	-	V	V
hsa04151:PI3K-Akt signaling pathway	-	V	V	V
hsa04152:AMPK signaling pathway	-	-	V	V
hsa04910:Insulin signaling pathway	-	V	V	-
hsa04922:Glucagon signaling pathway	-	-	V	-
hsa04931:Insulin resistance	-	-	V	-

^1^ Head kidney.

**Table 6:** Human diseases related to the GO analysis in common carp.

Disease Name	Brain	Gill	HK ^1^	Liver	Intestine
Autistic Disorder	V	-	-	-	-
Bipolar Disorder	V	-	-	-	-
Convulsions	V	-	-	-	-
Depressive Syndrome	V	-	-	V	-
Epilepsy	V	-	-	-	-
Manic	V	-	-	-	-
Mood Disorders	V	-	-	-	-
Neurodevelopmental Disorders	V	-	-	-	-
Schizophrenia	V	-	-	-	-
Liver Carcinoma	-	V	V	V	-
Breast Carcinoma	-	V	-	V	-
Colorectal Neoplasms	-	V	-	-	-
Q Fever	-	-	V	-	-
Diabetes	-	-	-	V	V
Heart failure	-	-	-	V	V
Obesity	-	-	-	V	-
Adenocarcinoma	-	-	-	-	V

^1^ Head kidney.

**Table 7:** Human diseases related to KEGG analysis in common carp.

Disease Name	Brain	Gill	HK ^1^	Liver	Intestine
Autistic Disorder	V	-	-	-	-
Bipolar Disorder	V	-	-	-	-
Convulsions	V	-	-	-	-
Depressive Syndrome	V	-	-	-	-
Epilepsy	V	-	-	-	-
Manic Disorder	V	-	-	-	-
Memory Disorders	V	-	-	-	-
Mood Disorders	V	-	-	-	-
Schizophrenia	V	-	-	-	-
Liver Carcinoma	-	V	V	V	-
Glycogen Storage Disease	-	V	V	-	V
Colorectal Neoplasms	-	V	-	-	-
Diabetes	-	-	-	V	V
Breast Carcinoma	-	-	-	V	-
Dementia	-	-	-	V	-
Alzheimer’s Disease	-	-	-	V	-
Pancreatic Neoplasm	-	-	-	V	-
Thyroid carcinoma	-	-	-	-	V

^1^ Head kidney.

## Data Availability

In this study, original sequencing data can be downloaded from the NCBI BioProject (submission ID PRJNA822546), and other data can be downloaded from supplementary data.

## Supplementary Materials

The following supporting information can be downloaded at: https://www.mdpi.com/article/10.3390/genes13061096/s1, Figure S1: GO similarity network analysis of common carp head kidney (HK), liver, and intestine under hypoxic conditions; Figure S2: Expression patterns of glycolysis/gluconeogenesis pathway in four common carp organs; Figure S3: Expression patterns of cell cycle pathway in the common carp gill; Figure S4: Expression patterns of DNA replication in the common carp gill; Table S1: Gene expression values under hypoxic conditions and normoxia in the brain, gill, head kidney (HK), liver, and intestine of common carp; Table S2: GO BP enrichment analysis by DAVID based on the DEGs with fold-change values up; Table S3: GO BP enrichment analysis by DAVID based on the DEGs with fold-change values down; Table S4: GO term descriptions from Figure S3; Table S5: GO term descriptions from Figure S4; Table S6: Gene expression involved in cholesterol metabolism-related GO functions in the common carp head kidney (HK) treated in hypoxia; Table S7: Gene expression involved in GO functions in the common carp brain treated in hypoxia; Table S8: KEGG pathway enrichment analysis by DAVID based on the DEGs with fold-change values up; Table S9: KEGG pathway enrichment analysis by DAVID based on the DEGs with fold-change values down; Table S10: Human disease enrichment analysis by DisGeNET through DAVID using genes collected from the GO enrichment analysis (including up and downregulated genes) of hypoxia-treated common carp; Table S11: Human disease enrichment analysis by DisGeNET through DAVID using genes collected from the KEGG pathway enrichment analysis (including up and downregulated genes) of hypoxia-treated common carp.
