# Supplementary material for: Comparative Transcriptome Analysis of Organ-Specific Adaptive Responses to Hypoxia Provides Insights to Human Diseases
Source: Genes (Basel). 2022 Jun 19;13(6):1096. doi: 10.3390/genes13061096 (PMC9222487; doi:10.3390/genes13061096)
Supplement: Supplementary file 1 [file genes-13-01096-s001.zip › 0.Supplementary Data.pdf]

Supplementary Data

Figure S1: GO similarity network analysis of common carp head kidney (HK), liver, and intestine under hypoxia: Each of the 3 similarity scores and 3 association scores was computed for each GO term pairs and a bubble network map was created.

Figure S2: Expression patterns of Glycolysis/Gluconeogenesis pathway in four common carp organs: All target genes are colored in gold and the organ-specificity is shown at in different points (gray: gill, orange: head kidney (HK), coffee red: liver, and blue: intestine.)

Figure S3: Expression patterns of Cell Cycle pathway in the common carp gill: All target genes are colored in navy blue.

Figure S4: Expression patterns of DNA replication in the common carp gill: Expression patterns of DNA replication in common carp gill. All target genes are colored in navy blue.

Table S1: Gene expression values under hypoxia and normoxia in the brain, gill, head kidney (HK), liver, and intestine of common carp: The expression of each sequence was measured in triplicate, and the average values were applied to calculate the fold change (hypoxia over normoxia). LogFC: log 2 value of fold change. FDR: false discovery rate from a p-value.

Table S2: GO BP enrichment analysis by DAVID based on the DEGs with fold change values up: Significant DEGs from Table S1 were pooled and annotated using DAVID. The enriched GO categories were selected with Benjamini adjusted p-values < 0.05 calculated from DAVID and hypoxia related GO terms are presented in the table with the yellow background color. HK: Head Kidney.

Table S3: GO BP enrichment analysis by DAVID based on the DEGs with fold change values down: Significant DEGs from Table S1 were pooled and annotated using DAVID. The enriched GO categories were selected with Benjamini adjusted p-values < 0.05 calculated from DAVID and hypoxia related GO terms are presented in the table with the yellow background color. HK: Head Kidney.

Table S4: Go term descriptions from Figure 3.

Table S5: Go term descriptions from Figure 4.

Table S6: Gene expression involved in cholesterol metabolism-related GO functions in the common carp head kidney (HK) treated in hypoxia: Gene expression involved in cholesterol metabolism-related GO functions in the common carp head kidney (HK) treated in hypoxia.

Table S7: Gene expression involved in GO functions in the common carp brain treated in hypoxia.

Table S8: KEGG pathway enrichment analysis by DAVID based on the DEGs with fold change values up: Significant DEGs from Table S1 were pooled and annotated using DAVID. The enrich KEGG categories were selected with Benjamini adjusted p-values < 0.05

calculated from DAVID and hypoxia relative KEGG terms are presented in the table with the yellow background color.

Table S9: KEGG pathway enrichment analysis by DAVID based on the DEGs with fold change values down: Significant DEGs from Table S1 were pooled and annotated using DAVID. The enrich KEGG categories were selected with Benjamini adjusted *p*-values < 0.05 calculated from DAVID and hypoxia relative KEGG terms are presented in the table with the yellow background color.

Table S10: Human disease enrichment analysis by DisGeNET through DAVID using genes collected from the GO enrichment analysis (including up- and down- regulated genes) of hypoxia treated common carp: The enriched human disease categories were selected with Benjamini adjusted *p*-values < 0.05 calculated from DAVID and hypoxia relatied disease terms are presented in the table with the yellow background color. HK: Head Kidney.

Table S11: Human disease enrichment analysis by DisGeNET through DAVID using genes collected from the KEGG pathway enrichment analysis (including up- and down-regulated genes) of hypoxia treated common carp: The enriched human disease categories were selected with Benjamini adjusted *p*-values < 0.05 calculated from DAVID and hypoxia related disease terms are presented in the table with the yellow background color. HK: Head Kidney.
