# Supplementary figures and images for: Comparative Transcriptome Analysis of Organ-Specific Adaptive Responses to Hypoxia Provides Insights to Human Diseases
Source: Genes (Basel). 2022 Jun 19;13(6):1096. doi: 10.3390/genes13061096 (PMC9222487; doi:10.3390/genes13061096)

## Slide 1
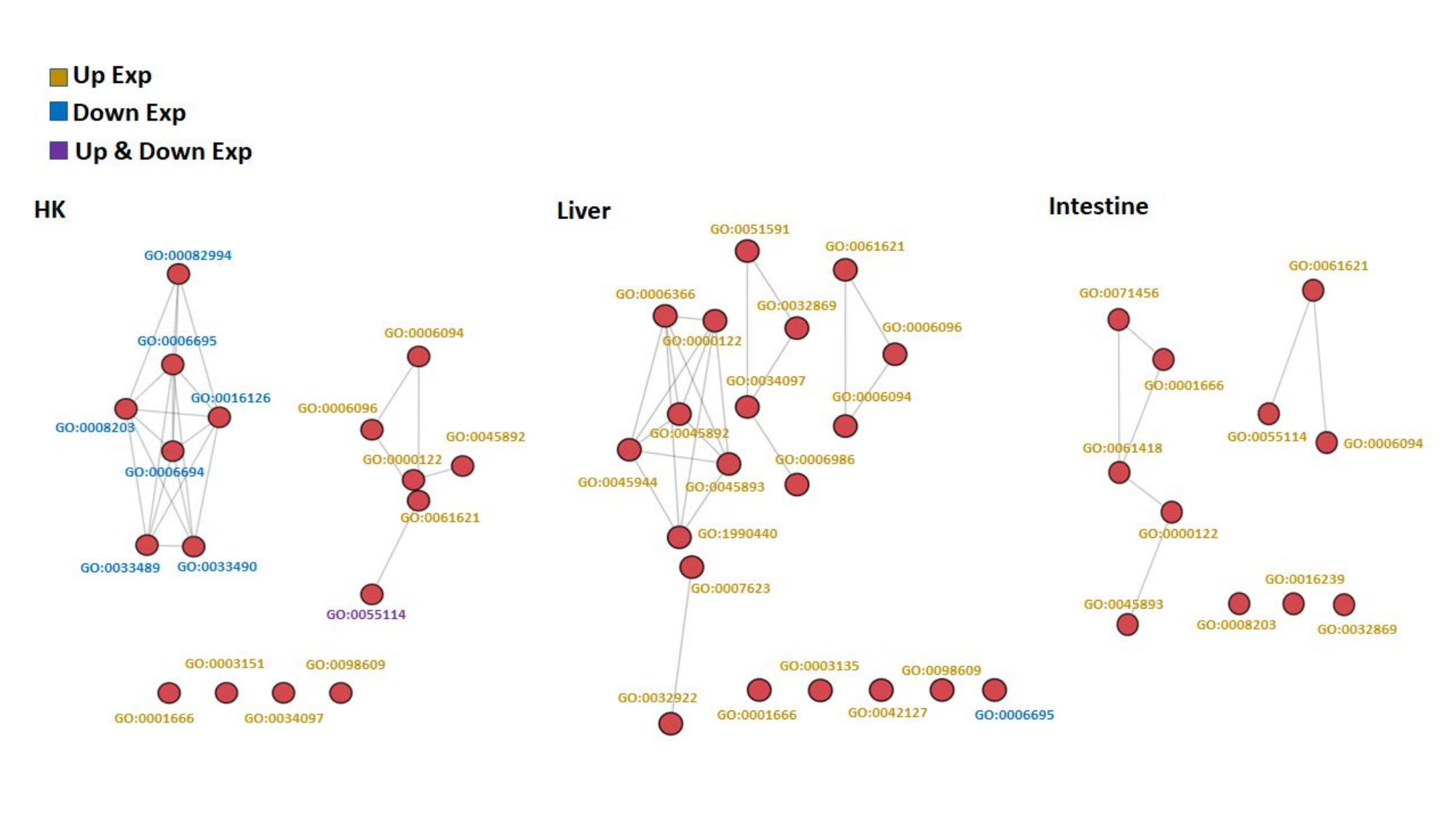

Supplement: Supplementary file 1 [file genes-13-01096-s001.zip › Figure S1.pptx]

## Slide 1
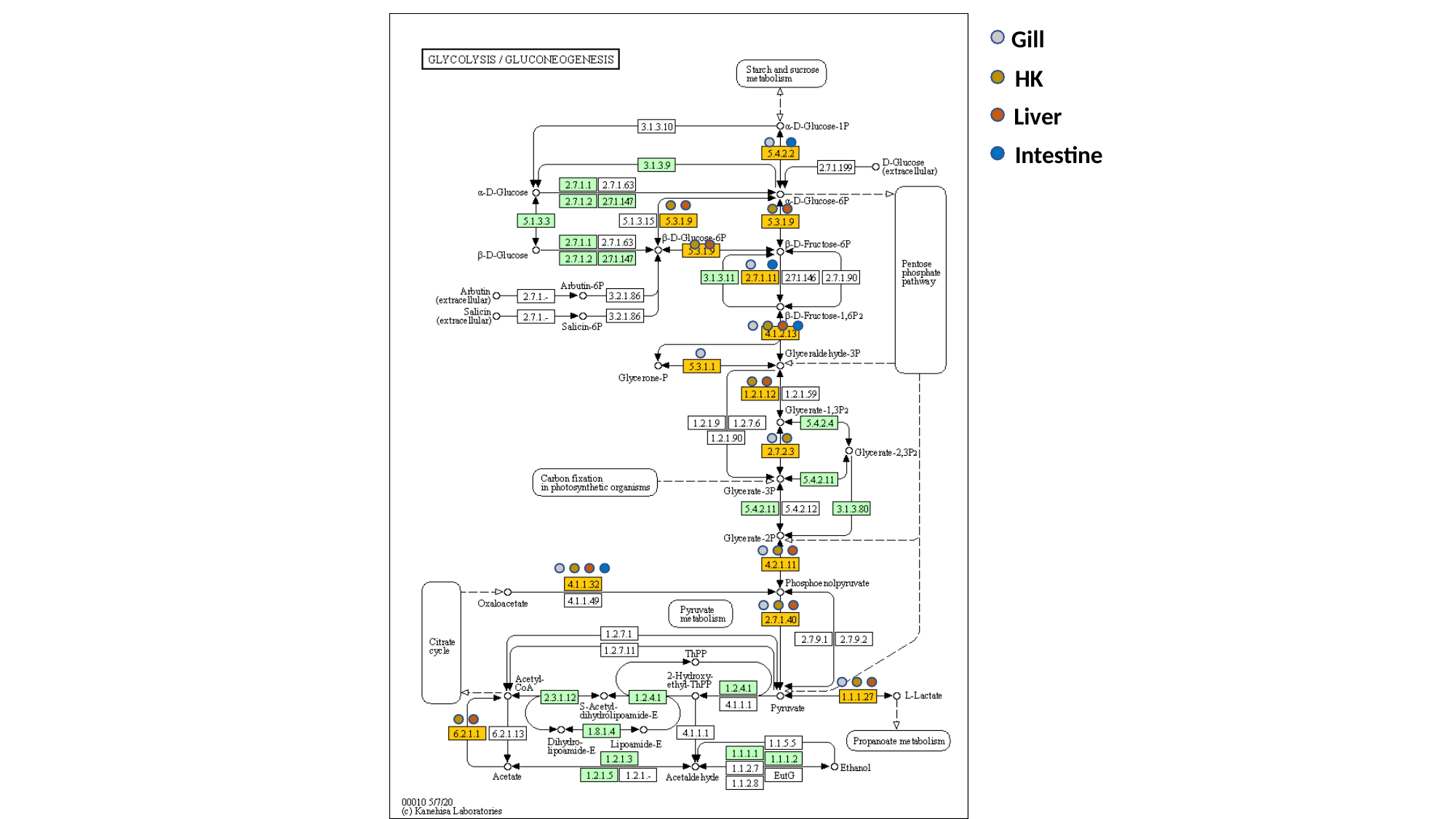

Gill
HK
Liver
Intestine

Supplement: Supplementary file 1 [file genes-13-01096-s001.zip › Figure S2.pptx]

## Slide 1
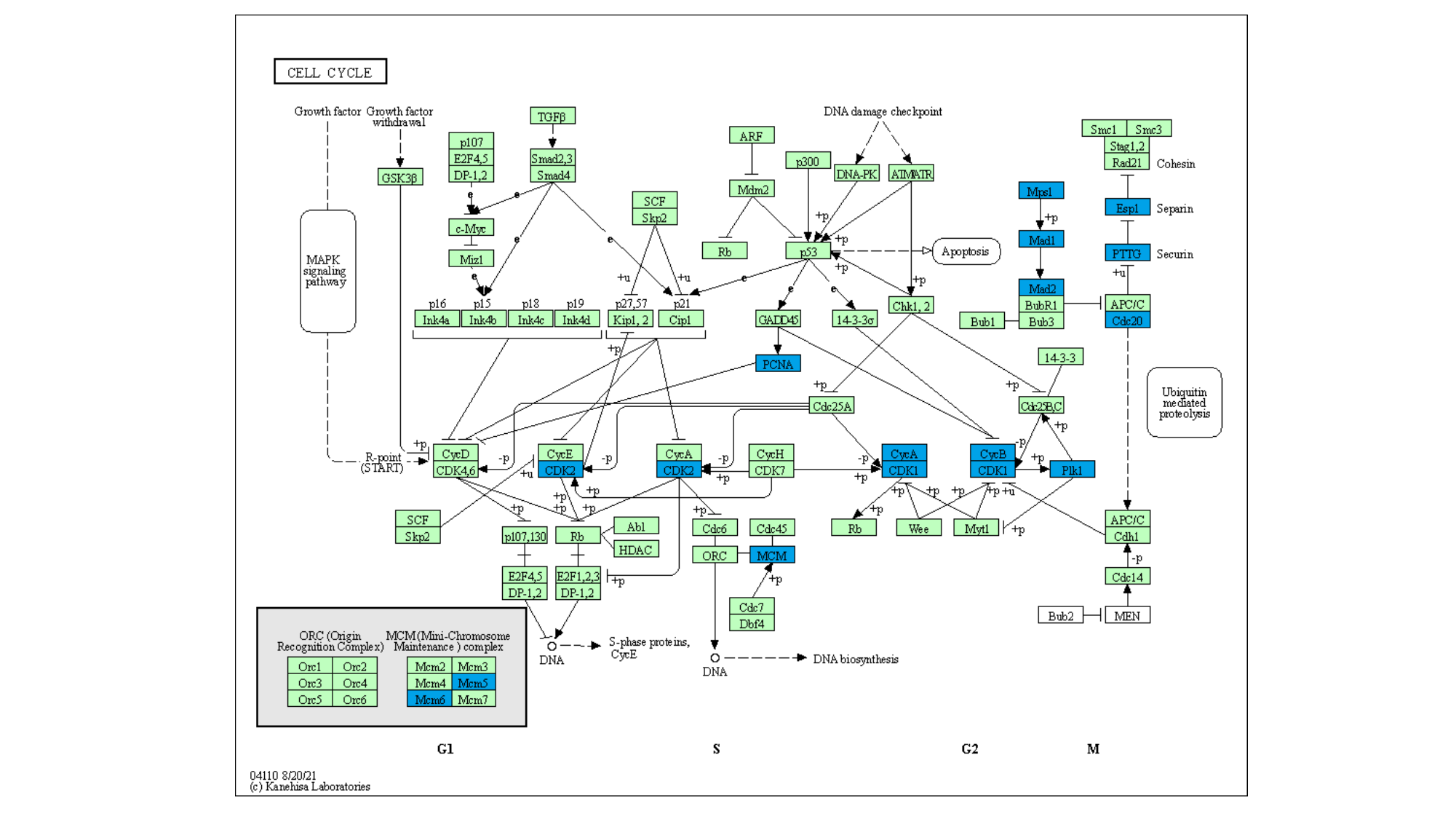

Supplement: Supplementary file 1 [file genes-13-01096-s001.zip › Figure S3.pptx]

## Slide 1
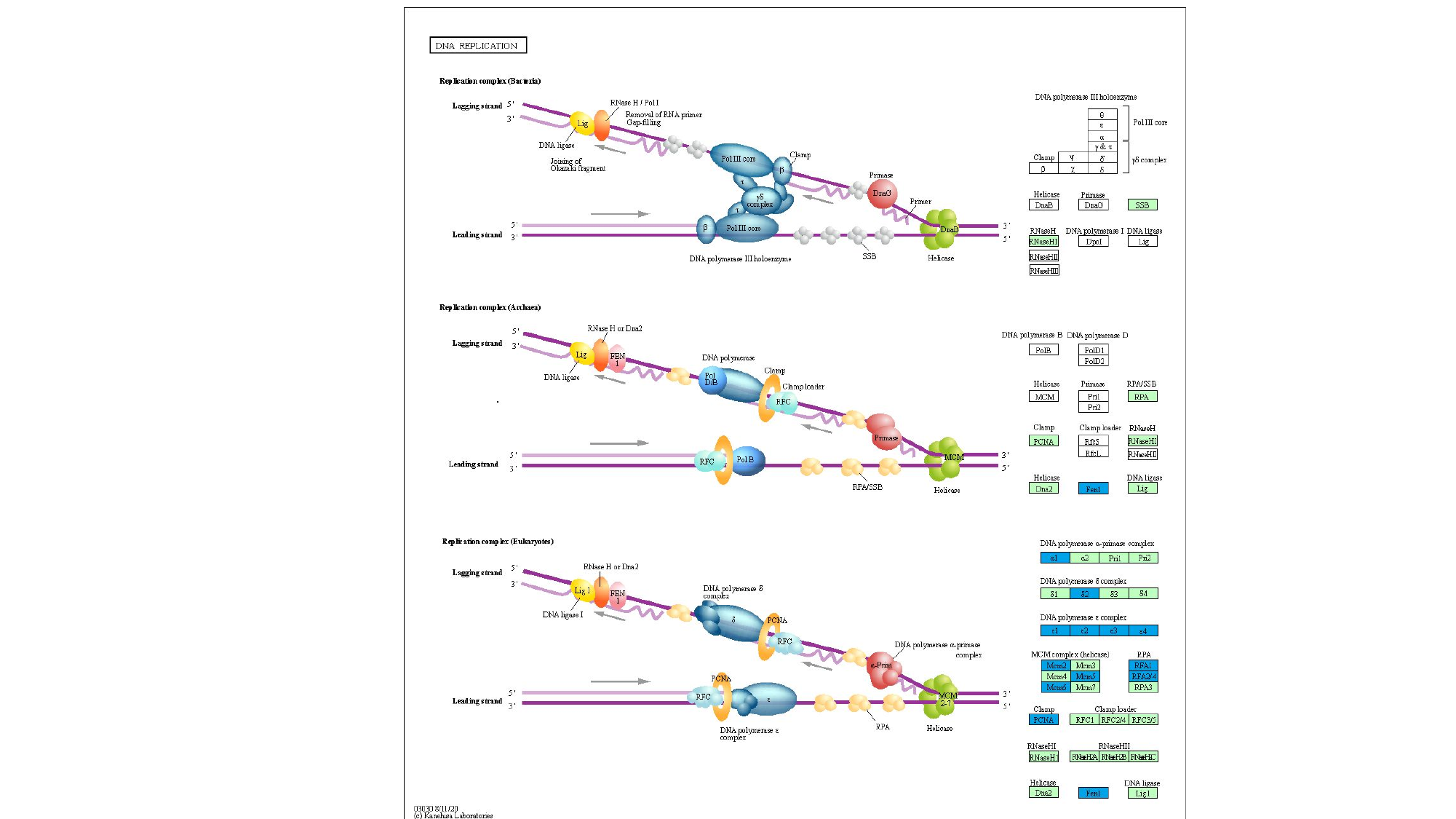

Supplement: Supplementary file 1 [file genes-13-01096-s001.zip › Figure S4.pptx]
